# Rare head and neck myositis with reversible myelodysplastic syndrome: The first reported lupus manifestation as an initial symptom

**DOI:** 10.5339/qmj.2025.65

**Published:** 2025-06-30

**Authors:** Ayu Paramaiswari, Muhammad Fakhrur Rozi, Gede Perdana Putera, Kartika Widayati

**Affiliations:** 1 Division of Rheumatology, Department of Internal Medicine, Faculty of Medicine Universitas Gadjah Mada, Yogyakarta, Indonesia; 2 Department of Internal Medicine, Faculty of Medicine Universitas Gadjah Mada, Yogyakarta, Indonesia; 3 Division of Hematology and Medical Oncology, Department of Internal Medicine, Faculty of Medicine, Universitas Gadjah Mada, Yogyakarta, Indonesia *Email: muhammadfakhrurrozi1993@mail.ugm.ac.id

**Keywords:** Autoimmune, systemic, myositis, immunosuppressants, steroid

## Abstract

**Introduction:**

Systemic lupus erythematosus (SLE) is a systemic autoimmune disease characterized by a dysregulated immune response against self-antigen, leading to multi-organ involvement. Myositis, as an initial manifestation of SLE, is a rare clinical entity, particularly in newly diagnosed patients.

**Case Presentation:**

A 27-year-old male presented with massive head and neck swelling, initially suspected to be superior vena cava syndrome (SVCS). Other symptoms included non-scarring alopecia, prolonged fever, oral ulcers, a history of hyperpigmented skin lesions, and progressive lower extremity weakness with edema. Hematological findings revealed persistent pancytopenia (anemia, leukopenia, and thrombocytopenia). Laboratory investigations demonstrated elevated muscle injury markers, including aspartate aminotransferase predominance and elevated creatine kinase. Immunological analysis showed a negative antinuclear antibody by indirect immunofluorescence, high anti-dsDNA titers, and normal complement levels. Bone marrow biopsy revealed trilineage dysplasia with macrophage activation, suggesting underlying hematologic involvement. Contrast-enhanced head and neck computed tomography ruled out SVCS, showing only diffuse muscle and subcutaneous edema. Based on the constellation of clinical, hematological, and imaging findings, the patient was diagnosed with myositis-associated SLE. The therapeutic approach included total plasma exchange (TPE), high-dose corticosteroid pulse therapy, and immunosuppressive induction therapy. Within 1 month of hospitalization, the patient demonstrated significant clinical and laboratory improvement and was subsequently transitioned to maintenance therapy with hydroxychloroquine (200 mg once daily), methylprednisolone (8 mg daily in a tapering regimen), and mycophenolate mofetil (500 mg twice daily). The patient achieved a lupus low disease activity state at follow-up.

**Discussion:**

This case represents a unique presentation of head and neck myositis in a newly diagnosed SLE patient, a manifestation not previously described in the literature. While orbital myositis in SLE has been reported, extensive myositis involving the head and neck as an initial SLE manifestation remains undocumented. Combining TPE, high-dose corticosteroids, and immunosuppressants was critical in disease control. Early recognition and aggressive immunomodulatory therapy are essential in managing such rare and severe SLE presentations.

**Conclusion:**

This case highlights an uncommon initial manifestation of SLE, emphasizing the importance of early clinical suspicion, comprehensive immunological and hematological evaluation, and prompt intervention. A multimodal therapeutic approach, including steroid pulse therapy, induction immunosuppression, and TPE, can lead to favorable clinical outcomes in severe and atypical SLE presentations.

## INTRODUCTION

Systemic lupus erythematosus (SLE) is a complex autoimmune disease with a highly variable presentation, ranging from asymptomatic cases to severe, refractory disease. Its pathogenesis involves intricate immunological dysregulation, primarily affecting T cells, B cells, and myeloid lineages. The development of SLE is commonly described using a three-stage autoimmune model: an initial phase of silent autoimmunity, followed by autoimmune reactivity, and culminating in clinically established SLE.^1,2^ Each stage is influenced by genetic and environmental factors, shaping distinct phenotypic and serologic characteristics. While antinuclear antibody (ANA) positivity is a hallmark of SLE, some patients with negative ANA-IF results may still meet the disease criteria through alternative autoantibodies, such as anti-dsDNA, SS-A/Ro 52, nucleosome, or SS-A/Ro 60.^3^

Myositis is an uncommon initial manifestation of newly diagnosed SLE, with a reported overall prevalence ranging from 5.6% to 7.3%.^4–6^ It is often associated with significant morbidity and mortality, resembling primary inflammatory myopathies. The rarity of lupus myositis, especially as a presenting feature in male patients, poses a diagnostic challenge and requires careful clinical and serological assessment. This case report highlights a male patient with SLE presenting with myositis as an initial symptom, an atypical and rarely documented occurrence. The following sections will discuss the diagnostic challenges and therapeutic considerations, contributing to a broader understanding of lupus myositis. Written informed consent was obtained from the patient to publish clinical data and images.

## CASE PRESENTATION

**Figure 1:**
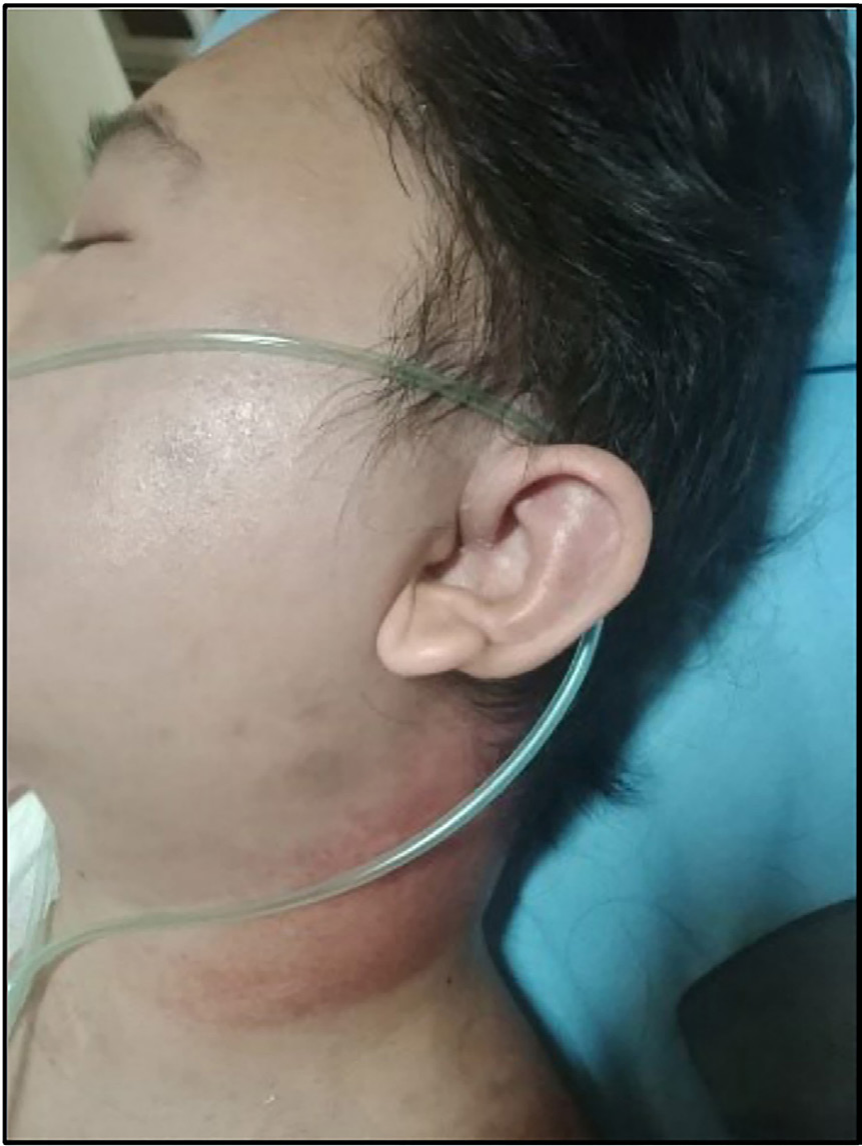
Pathognomonic findings associated with myositis: redness in the neck region represented tissue inflammation, referred to as the “shawl sign.”

**Figure 2:**
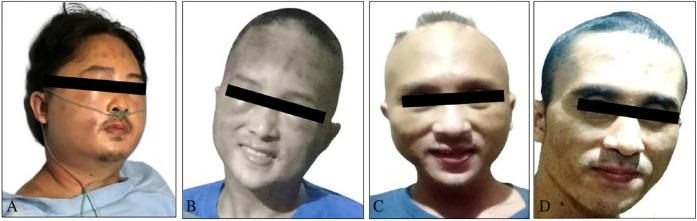
The clinical picture of patient symptom resolution following several therapies. (A) Patient on the 20th day of hospital admission, (B) At discharge period of recent hospital admission (TPE once weekly and 3-day MP pulse therapy), (C) 1-month post-acute therapy, (D) 2 months after discharge from hospital.

**Figure 3:**
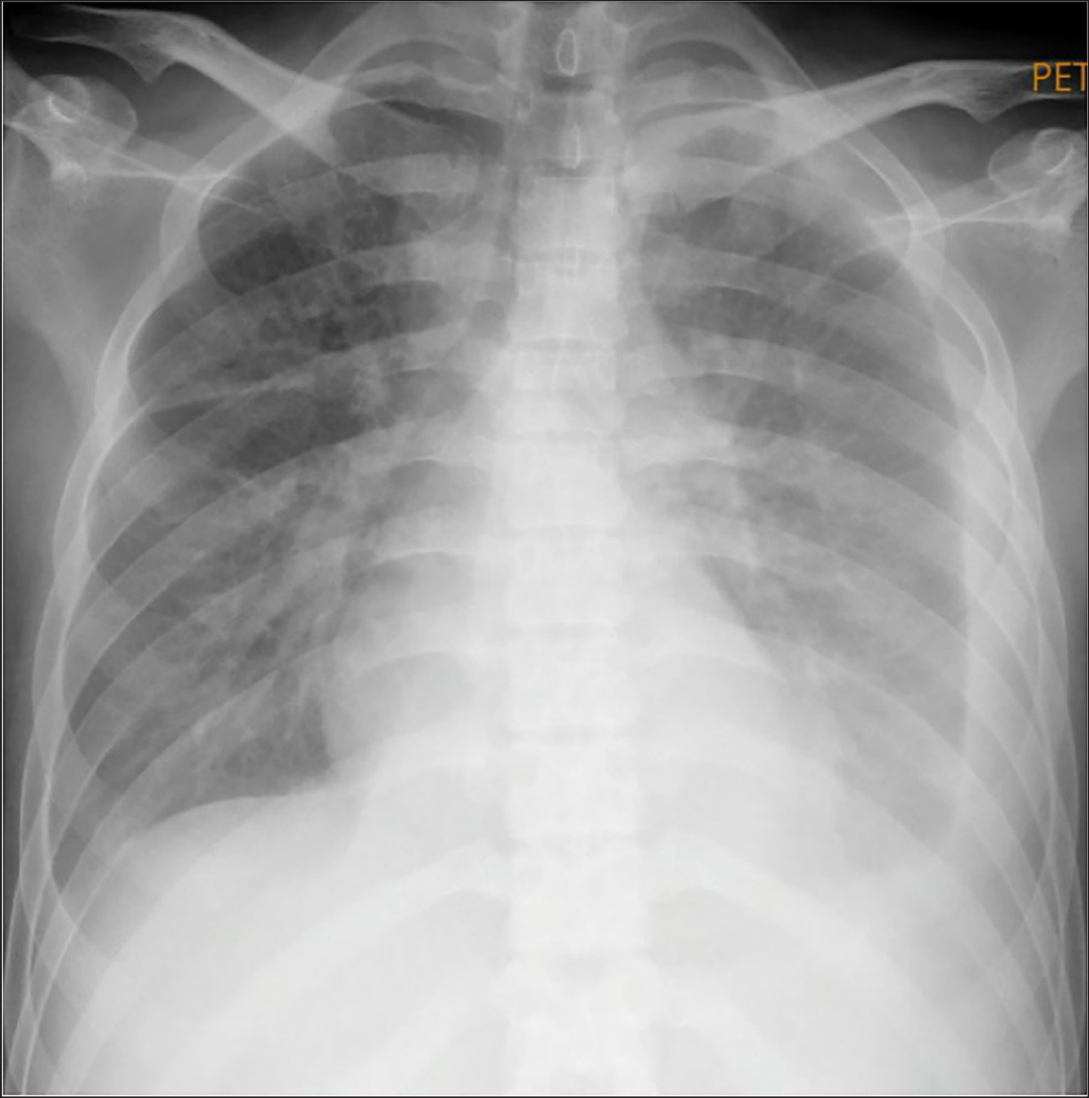
Chest radiographic findings demonstrated bilateral pleural effusion.

A 27-year-old male presented to the emergency room with a 2-week history of progressive head and neck swelling, which caused significant difficulty in swallowing and breathing (Figure 1). Additional symptoms included non-scarring alopecia, oral ulcers, fever, chronic non-productive cough, and an 8 kg weight loss over 3 months. Physical examination revealed marked facial and neck edema (Figure 2A), decreased tactile fremitus on the left hemithorax, suggesting pleural effusion, and significantly reduced motor power (2/2) in both lower extremities. Sensory function remained intact. Chest radiography confirmed bilateral pneumonia and a left-dominant pleural effusion (Figure 3). Electrocardiography showed no abnormalities, while echocardiography demonstrated mild pericardial effusion with preserved cardiac function. The patient also reported progressive lower limb weakness persisting for a month (motor power 2/2; sensory function remained intact).

A hematologic evaluation revealed pancytopenia with anemia (10.2 g/dL), thrombocytopenia (30,000 cells/μL), leukopenia (2,000 cells/μL), lymphocytopenia (210 cells/μL), and a low absolute neutrophil count (1,710 cells/μL). Liver enzyme abnormalities showed elevated aspartate aminotransferase (AST) at 331 U/L, exceeding alanine aminotransferase at 202 U/L with accompanying elevated lactate dehydrogenase (LDH) (2,080 U/L) and creatine kinase (CK) (1,483 U/L) suggesting muscle injury. Immunologic tests showed negative ANA-IFF and ANA test, normal complement levels (C3: 132 mg/dL, C4: 38 mg/dL), and high anti-dsDNA (32.4 IU/L).

The diagnosis of SLE was established based on the ACR 1997 and SLICC 2012 criteria, as the patient met five clinical criteria: mucocutaneous (alopecia and oral ulcers), serositis (pleural and pericardial effusion), suspected myositis (elevated CK and muscle edema on imaging), constitutional symptoms (fever and weight loss), and hematologic abnormalities (pancytopenia). These findings resulted in a SLEDAI-2K score of 17, indicating severe disease activity. However, the patient failed to meet the ACR/EULAR 2019 classification criteria, as a positive ANA-IFF is a mandatory entry criterion. The absence of low complement levels and antiphospholipid antibodies also precluded alternative classification under the 2021 guidelines. Nevertheless, the constellation of clinical features and the presence of high anti-dsDNA.

**Figure 4:**
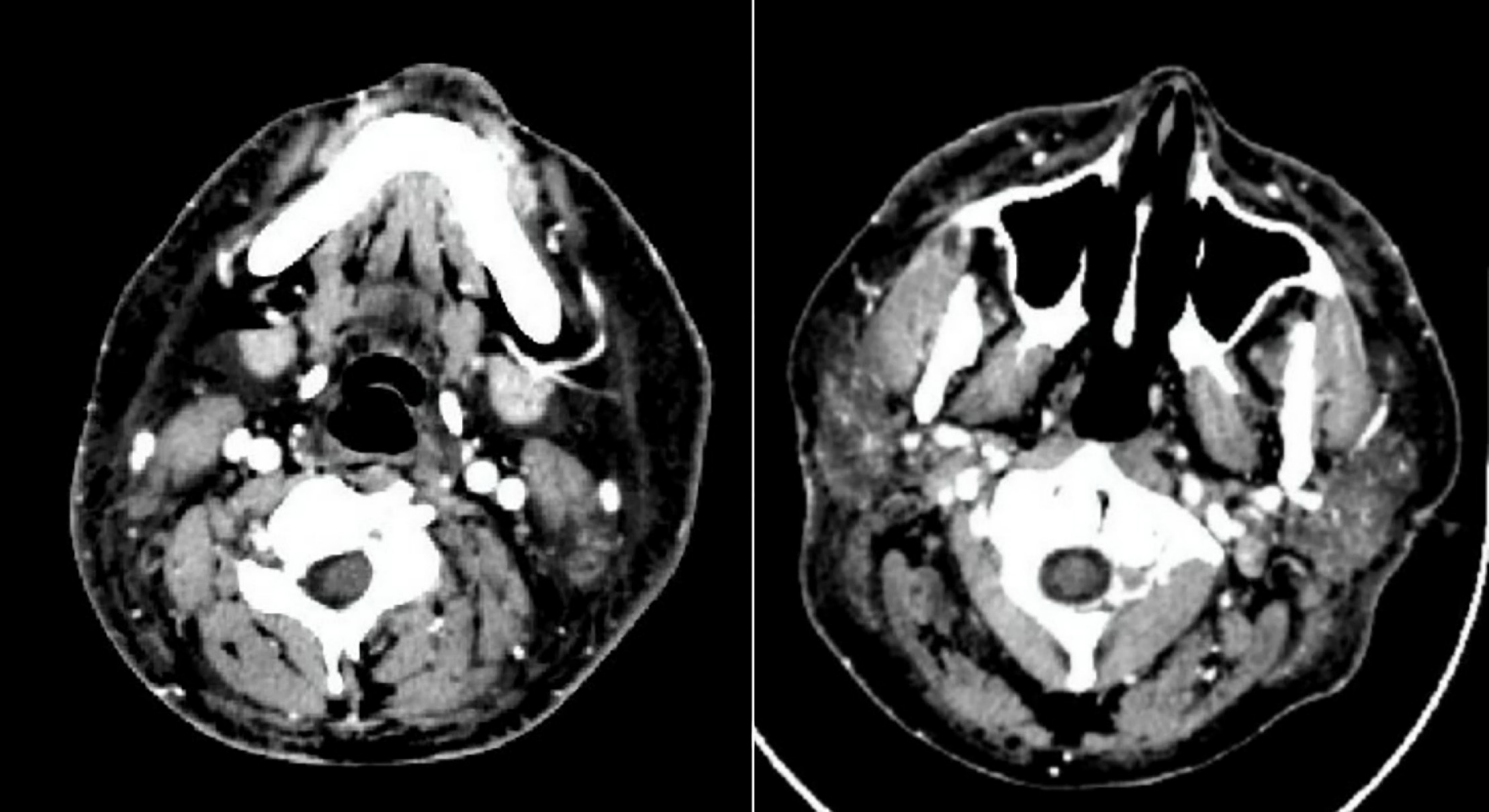
A contrast head and neck CT scan (axial view) confirming no mass or thrombus, yet subcutis and muscle swelling in the head and neck regions were evident.

The diagnosis of myositis-related SLE was confirmed through a combination of clinical, biochemical, and imaging findings. The patient reported progressive lower extremity weakness for 1 month, corresponding with significantly elevated CK levels and LDH, indicative of muscle injury. Contrast-enhanced computed tomography (CT) imaging of the head and neck revealed diffuse subcutaneous and muscle edema, ruling out vascular compression syndromes such as VCSS (Figure 4). These findings were consistent with an inflammatory myositis process rather than an infectious or thrombotic cause. Bone marrow aspiration demonstrated dyserythropoiesis and dysthrombopoiesis, with macrophage activation suggestive of an underlying autoimmune-mediated bone marrow dysfunction. This finding was consistent with secondary myelodysplastic syndrome (MDS) associated with SLE, and myositis was established as the final diagnosis.

In the first phase of hospitalization (days 1–7), the patient was treated with levofloxacin 750 mg once daily for pneumonia, ibuprofen 400 mg three times daily, ursodeoxycholic acid 250 mg three times daily, methylprednisolone 62.5 mg once daily, and hydroxychloroquine (HCQ) 200 mg once daily. On the 8th day, cyclosporin (100 mg twice daily) was introduced following infection resolution but later switched to mycophenolate mofetil (MMF) 500 mg twice daily due to a bilirubin surge (from 0.94 mg/dL at admission to 4.12 mg/dL within 1 week of cyclosporin use). Total plasma exchange (TPE) was initiated every 4 days (from days 10 to 21) as a supplementary strategy, followed by high-dose pulse methylprednisolone (750 mg/day for 3 days, starting on day 22), leading to marked clinical improvement.

By day 26, the patient demonstrated significant recovery, including a substantial increase in platelet count (from 15,000 cells/μL on day 6 to 133,000 cells/μL at discharge), normalization of absolute neutrophil count (from 360 to 2,990 cells/μL), improved lower extremity motor function (5/5), and a reduction in muscle injury markers (CK decreased from 1,483 to 22 U/L). The edematous swelling of the head and neck showed remarkable resolution (Figures 2B–D). Induction immunosuppressive therapy was continued for 4 months post-diagnosis of myositis and MDS, ensuring sustained disease control.

## DISCUSSION

SLE presents a broad spectrum of clinical manifestations, complicating diagnosis and management. Myositis and MDS are rare initial manifestations of SLE, posing additional diagnostic and therapeutic challenges. This case highlights the complexity of diagnosing and managing an SLE patient with atypical immune profiles and severe systemic involvement. Management strategies involve various approaches, including infection control, plasma exchange, immunosuppressant, and pulse steroid administration. Controlling infection as the primary hit in SLE-provoking immune perturbation remains a main therapeutic approach before central SLE management utilizing immunosuppressants and steroids.^7^

SLE diagnosis relies on classification criteria, with the ACR/EULAR 2019 scoring system being the most sensitive and specific.^8,9^ However, a positive ANA-IFF result is required as an entry criterion, which this patient lacked. While alternative entry criteria in the 2021 SLE guidelines allow clinical SLE diagnosis based on low complement and antiphospholipid antibodies, the absence of these markers further complicated classification.^10^ Despite this, clinical features such as persistent pancytopenia, fever of unknown origin, undocumented skin lesions, mucosal ulcers, and alopecia strongly suggested an underlying autoimmune process. Consequently, high anti-dsDNA, a marker with high specificity but low sensitivity, played a pivotal role in diagnosis.^11^

Two main facades in this SLE patient are myositis and hematologic manifestation. Myositis in SLE is uncommon, occurring in less than 11% of cases, and is more frequently associated with overlap syndromes like dermatomyositis or polymyositis. Focal myositis affecting the head and neck is exceedingly rare, with only seven reported cases of orbital myositis responsive to high-dose steroids.^12–14^ To date, no documented cases describe massive head and neck swelling secondary to SLE myositis. This case represents the first reported instance of head and neck myositis as the initial SLE manifestation, underscoring the importance of recognizing atypical presentations.

Hematologic abnormalities are common in SLE, with anemia, leukopenia, and thrombocytopenia frequently observed due to autoimmune-mediated destruction. This patient exhibited pancytopenia with a hemolytic pattern on peripheral blood smear. However, bone marrow biopsy revealed two-lineage dysplasia, suggesting secondary MDS rather than classic SLE-associated cytopenia. While MDS-SLE is rare, studies indicate its association with severe disease phenotypes, including neuropsychiatric and renal involvement, which were absent in this patient.^15^ The underlying mechanism remains unclear, though reversible bone marrow dysplasia has been reported in SLE patients, potentially linked to disease severity.^16^

Management of severe SLE requires a multimodal approach tailored to disease activity and complications. HCQ remains the cornerstone of treatment in newly diagnosed SLE. For other immunosuppressants, this patient was initiated on cyclosporin and later switched to MMF to mitigate hepatobiliary toxicity following an acute bilirubin increase. High-dose steroids are essential for suppressing inflammation, and this patient received 3 days of pulse methylprednisolone followed by maintenance therapy. Infection remains a major concern in SLE, both as a disease trigger and a complication of immunosuppressive therapy, requiring careful adjustment of immunosuppressant timing based on infection resolution.^17^ As adjunctive therapy, plasma exchange was utilized to reduce circulating autoantibodies, facilitating disease control rapidly.^18^

With this comprehensive approach, the patient achieved a low lupus disease activity state, demonstrating the efficacy of timely and individualized treatment strategies in complex SLE presentations. This case reinforces the importance of recognizing atypical manifestations and adapting therapeutic regimens accordingly.

## CONCLUSION

SLE is a complex autoimmune disease with diverse clinical presentations. Its diagnosis can be particularly challenging due to atypical immunologic profiles and overlapping manifestations with other conditions. The disease can involve multiple organ systems, requiring careful evaluation to guide diagnosis and treatment. In rare cases, myositis and MDS may serve as the initial manifestations of SLE, potentially delaying diagnosis, particularly when accompanied by atypical immunologic findings. This diagnostic challenge underscores the importance of considering autoimmune diseases when signs and symptoms affect at least two organ systems. Early recognition is crucial, as young males and females may present with non-classical features that warrant further autoimmune evaluation. Following a definitive diagnosis, timely intervention is essential to control systemic inflammation and achieve remission. A multimodal therapeutic approach, including high-dose corticosteroids, immunosuppressive agents, and plasma exchange, can be employed to optimize patient outcomes. Therefore, clinicians should maintain a high index of suspicion for atypical presentations of SLE and initiate prompt, targeted treatment to improve prognosis.

## Conflicts of interest

None.
